# Female virilization related to congenital adrenal hyperplasia and psychological distress

**DOI:** 10.1192/j.eurpsy.2024.1217

**Published:** 2024-08-27

**Authors:** B. Abdelmoula, N. Ramma, M. Ben Yedder, I. Bouaziz, N. Bouayed Abdelmoula

**Affiliations:** Genomics of Signalopathies at the service of Precision Medicine - LR23ES07, Medical University of Sfax, Sfax, Tunisia

## Abstract

**Introduction:**

In females, congenital adrenal hyperplasia (CAH), a spectrum of inherited genetic conditions related to the disruption of adrenal steroidogenesis, is among the most common conditions leading to inappropriate virilization. For adolescent and adult women, progression of hirsutism may have many psychological concerns.

**Objectives:**

To explore the psychological distress of a young Tunisian woman who sought medical help and psychological support at a late stage, after suffering from genital ambiguity and severe virilization.

**Methods:**

Harboring phenotypic male transformation at puberty, our patient attended genetic counselling for cytogenetics assessment. Clinical, biological, psychological and genetic explorations were thus carried out.

**Results:**

A 17-year-old female was born from first-degree consanguineous parents, and had healthy siblings (a sister and three brothers). After a single menstrual episode at puberty, she developed amenorrhea and an unexpected progressive virilization, including hirsutism with an inappropriate beard that she had to shave every day and a male voice. Clinical examination revealed a male morphotype with an enlarged clitoris that resemble a penis, male-type pubic hair, underdeveloped of breasts, abnormal cutaneous hyperpigmentation, and a short stature. Pelvic ultrasound revealed a small uterus, but with no visualized gonads. Genetic exploration showed a female 46,XX karyotype and the absence of Y chromosome sequences. Diagnosis of a non-classic CAH was confirmed. Psychological assessment found that the psychological development of the sexual identity corresponded to the assignment of the female sex. A severe psychological suffering due to the non-acceptance of her virile appearance impaired the quality of her daily personal and social life. Stigmata of a depressive syndrome were also revealed.

**Conclusions:**

Particular attention to the psychological assessment of patients with CAH is recommended, as changes in physical appearance have a detrimental impact on psychological and mental well-being.

**Disclosure of Interest:**

None Declared

